# Evaluating the Effects of Stress, Depression, and Anxiety on Hypertension

**DOI:** 10.7759/cureus.86077

**Published:** 2025-06-15

**Authors:** Nenrot S Gopep, Saliu A Shittu, Okelue E Okobi, Chekwube M Obianyo, Zimakor D Ewuzie, Christy N Anyaogu, Precious A Uzoh, Ifunanya P Arinze, Maureen C Eze

**Affiliations:** 1 Community Medicine, Federal Medical Center, Keffi, NGA; 2 Public Health, Georgia Southern University, Statesboro, USA; 3 Nephrology, Oasis Kidney Care Center, Houston, USA; 4 Family Medicine, Larkin Community Hospital Palm Springs Campus, Miami, USA; 5 Family Medicine, Lakeside Medical Center, Belle Glade, USA; 6 General Medicine, Jiann-Ping Hsu College of Public Health, Georgia Southern University, Statesboro, USA; 7 Adult Psychiatry, Cygnet Hospital Harrogate, Harrogate, GBR; 8 Internal Medicine, Richmond Gabriel University, Kingstown, VCT; 9 Family Medicine, Senator's Health Center Family Medicine Collaboration, Glace Bay, CAN; 10 Family Medicine, Nova Scotia Health Authority, Glace Bay, CAN; 11 General Medicine, Ternopil National Medical University, Ternopil, UKR; 12 General Medicine, Abia State University, Uturu, NGA

**Keywords:** anxiety, cardiovascular risk, depression, hypertension, mental health, multiple imputation, stress

## Abstract

Introduction: Hypertension is a persistent global health issue, with psychological factors like stress, anxiety, and depression increasingly studied as potential contributors. However, their independent associations with hypertension remain unclear.

Objective: To examine the relationship between stress (using self-reported irritability as a proxy), anxiety, and depression with both the prevalence and severity of hypertension in a nationally representative adult population.

Methods: A cross-sectional analysis was conducted using publicly available data from the National Health and Nutrition Examination Survey (NHANES), a nationally representative survey conducted by the Centers for Disease Control and Prevention (CDC). The study included 18,891 adults aged 18 and older. Hypertension was defined by self-reported medical diagnosis. Psychological variables included depression and anxiety scores, with stress proxied by reported irritability. Covariates included age, gender, race/ethnicity, physical activity, and BMI. Due to missing data, multiple imputation by chained equations (MICE) was applied. Multivariable logistic regression was used to assess associations.

Results: After adjustment, stress was significantly linked to higher odds of hypertension (β = 0.30, p < 0.03), while anxiety and depression were not. Older age, higher body mass index (BMI), and physical inactivity were also significant risk factors. Non-Hispanic Black and White participants had greater odds of hypertension compared to Mexican Americans.

Conclusion: Stress was independently associated with hypertension, emphasizing the relevance of emotional strain in cardiovascular risk. Anxiety and depression showed no significant association. Findings support the inclusion of stress assessment in hypertension screening, though results are limited by the study's cross-sectional design and reliance on imputed data.

## Introduction

In the modern world, high blood pressure or hypertension (HTN) is a major risk factor for cardiovascular diseases and premature death across the globe. It is a chronic illness that affects millions of people globally, and its etiology extends beyond traditional physical factors like poor diet, inactivity, and heredity [1]. Researchers as well as specialists in healthcare have started realizing the vital role that psychological health plays in the development and course of hypertension. Factors like stress, depression, and anxiety have been significantly associated with the onset and worsening of high blood pressure, thus calling for an urgent investigation of the same in a systematic and data-driven way [2-5].

Sleep quality, a dimension often undervalued in clinical practice, is among the significant psychosocial factors that affect stress. Geiker et al. [2] argue that difficulty sleeping, either in the form of insomnia, disrupted sleep, or poor sleep quality, may elicit a physiological stress response. Not getting enough sleep activates the hypothalamic-pituitary-adrenal (HPA) axis, which starts the release of cortisol and increases activity in the sympathetic nervous system. This sequence of occurrences in the biological reactions leads to vasoconstriction, which is accompanied by increased heart rate and blood pressure, which contributes to the onset and sustenance of hypertension. Chronic sleep deprivation is not only known to raise blood pressure during the day but also interfere with the normal nocturnal fall in blood pressure levels, which further exerts stress on cardiovascular health [2].

Psychological disorders, such as depression and anxiety, increase the stress response mechanisms in the body. Spytska [4] argues that depression can change autonomic nervous system regulation and can disturb lifestyle-related behaviors, such as exercise and medication compliance, which can then directly cause hypertension. Likewise, anxiety, in particular the chronic type, causes hyperstimulation of stress hormones like adrenaline and noradrenaline, subsequently increasing blood pressure and impairing vascular functioning [5]. These psychological disturbances can also amplify the perception of stressors, forming a vicious circle where physiological dysfunction feeds back into poor emotional regulation.

With the complexity of mental-cardiovascular health interplay, it becomes imperative to investigate this relationship scientifically through reliable data [6]. Even though we know that chronic diseases can cause psychological stress, it's important to study how psychological distress might lead to chronic diseases like hypertension in a more scientific way. Recent studies in the United States have highlighted a significant prevalence of anxiety and depression among adults [7-11]. Data from the National Health Interview Survey indicated that in 2022, approximately 18.2% of adults experienced symptoms of anxiety, while 21.4% reported symptoms of depression within a two-week period, with notably higher rates among adults aged 18-29 and women [12,13]. During the COVID-19 pandemic, adults aged 18 to 39 were disproportionately affected, exhibiting higher rates of anxiety (40%) and depression (33%) compared to older adults [14]. A meta-analysis of U.S. college students also reported pooled prevalence rates of 31% for anxiety symptoms and 34% for depression, reinforcing the mental health burden among younger populations [15,16]. According to the American Psychological Association's 2023 Stress in America survey, 64% of adults identified stress as a significant health concern, and 36% reported experiencing extreme stress in the past month [17]. Moreover, national data show that approximately 30% of adults score in the high range on the Perceived Stress Scale [18].

These findings underscore the urgency of addressing psychological distress as part of holistic approaches to chronic disease prevention. These findings collectively emphasize the growing concern of mental health issues among young adults in the U.S., which points to the importance of targeted interventions. The findings are expected to offer timely perspectives on preventive and integrative care measures that illustrate the importance of mental health screening and sleep hygiene in managing blood pressure and wellness. The objective of this study is to examine the relationship between stress (using self-reported irritability as a proxy), anxiety, and depression with both the prevalence and severity of hypertension in a nationally representative adult population.

## Materials and methods

Study design and data sources

This study employed a cross-sectional descriptive design utilizing publicly available data from the National Health and Nutrition Examination Survey (NHANES) spanning the 1999-2004 cycles due to data unavailability, with a sample size of 18,698 [6]. NHANES is a nationally representative survey conducted by the Centers for Disease Control and Prevention (CDC) that combines interviews, physical examinations, and laboratory testing to assess the health and nutritional status of the civilian, non-institutionalized U.S. population. The survey employs a complex, multistage probability sampling design, allowing generalization to the U.S. population.

This study was conducted using secondary analysis of publicly available, de-identified data from the National Health and Nutrition Examination Survey (NHANES). According to the U.S. Centers for Disease Control and Prevention (CDC), NHANES data do not contain identifiable private information, and therefore, Institutional Review Board (IRB) approval is not required for secondary analysis. In accordance with the U.S. Department of Health and Human Services’ Code of Federal Regulations (45 CFR 46), the International Council for Harmonisation (ICH) Good Clinical Practice (GCP) guidelines, the Declaration of Helsinki, and the Belmont Report, ethical approval is not required for studies using publicly available and anonymized data. This study adhered to all applicable ethical standards for research involving secondary data use.

Inclusion and exclusion criteria

This study utilized secondary data from NHANES [6], which includes pre-defined populations and variable structures based on standardized national protocols. All adults aged 18 years and older who completed the relevant mental health modules (anxiety, depression, and irritability) and had data available for hypertension status were included in the analysis.

Participants were excluded only if they lacked responses on variables critical to the study that could not be recovered through multiple imputations, that is, if they consisted of completely missing outcome data. Additionally, we excluded observations that were not relevant to the research objective, such as those related to specific subpopulations, e.g., children. The NHANES dataset documentation flagged the exclusion of pregnant individuals to prevent potential confounding due to physiological changes affecting blood pressure.

Variables and measurements

Hypertension (HTN) served as the outcome variable, defined by the self-reported history of a medical diagnosis of high blood pressure (yes/no). The main factors looked at were anxiety, measured with the generalized anxiety disorder-7 (GAD-7) scale, and depression, assessed using the Patient Health Questionnaire-9 (PHQ-9) scale; both are trusted tools for checking mental health in large groups of people.

Patient Health Questionnaire (PHQ-9): The PHQ-9 is a clinically validated tool frequently employed in both primary care and mental health settings to assess the severity of depressive symptoms [8]. It consists of nine items that correspond to the diagnostic criteria for major depressive disorder, with respondents indicating how often they experienced each symptom over the prior two weeks. Based on the total score, depression severity is categorized as follows: 0-4 (minimal), 5-9 (mild), 10-14 (moderate), 15-19 (moderately severe), and 20-27 (severe) [12].

Generalized Anxiety Disorder Scale (GAD-7): The GAD-7 is a widely used screening instrument designed to identify and evaluate the severity of generalized anxiety disorder and related conditions [7]. The questionnaire includes seven items reflecting core anxiety symptoms, with responses based on symptom frequency. The severity classifications are 0-4 (minimal), 5-9 (mild), 10-14 (moderate), and 15-21 (severe) [12].

In the absence of a direct stress measure, stress was proxied by self-reported irritability lasting at least two weeks, reflecting the emotional strain commonly associated with stress in research. Covariates included age (continuous), gender (male/female), race/ethnicity (Mexican American, other Hispanic, non-Hispanic White, non-Hispanic Black, and non-Hispanic Asian), physical activity (average daily level), and body mass index (BMI), calculated from measured height and weight.

Missing data and imputation approach

A substantial proportion of data was missing for key mental health variables, approximately 80% for GAD, DEP, and stress irritability. Additionally, key mental health modules had high nonresponse rates in NHANES 1999-2004 (due to nonparticipation in optional components). To fix these gaps and keep the number of participants, the study used multiple imputation by chained equations (MICE) with the mi impute chained command in Stata version 18 (StataCorp LLC, College Station, TX). A total of 20 imputations were generated. Imputation models were created using the right types of regression for continuous and binary variables, including all important predictors and covariates, to ensure the missing data is assumed to be random (MAR). We conducted all subsequent analyses on the imputed dataset using the mi-estimate commands, which account for both within- and between-imputation variability.

Statistical analysis

Using Stata version 18, descriptive statistics were calculated for all variables using pooled estimates across the 20 imputations. Finally, a multiple logistic regression was performed to assess the independent association of anxiety, depression, stress, and irritability with hypertension, adjusting for age, gender, race/ethnicity, physical activity, and BMI. All regression models were estimated using the mi-estimate (logistic) command to account for imputed data. All the findings from this study were subjected to a 5% significance level.

## Results

Descriptive statistics

Table 1 presents descriptive statistics that were calculated to summarize the characteristics of the study population. Key variables included hypertension status, mental health indicators (anxiety, depression, and stress), and demographic and lifestyle factors such as age, gender, race/ethnicity, physical activity, and body mass index (BMI).

**Table 1:** Descriptive statistics for the characteristics of the study population. HTN: hypertension; GAD: generalized anxiety disorder score; Stress_Irr: stress irritability; DEP: depression screening scale; PAQ: physical activity; BMI: body mass index; SD: standard deviation; Max: maximum; N: number.

Variable	Description	Mean	SD	Min	Max	N
HTN	Hypertension	0.27	0.45	0	1	18,395
GAD	Generalized anxiety disorder score	4.76	0.61	1	7.19	18,439
Stress_Irr	Stress irritability	0.57	0.45	0	1	18,438
DEP	Depression screening scale	4.12	1.09	0.22	8.14	18,439
Race	Race/ethnicity	2.75	1.15	1	5	18,698
Gender	Coded as male or female	0.58	0.49	0	1	18,692
Age	Age in years	49.90	17.81	19	85	18,692
PAQ	Physical activity	2.04	0.86	1	9	18,692
BMI	Body mass index	27.72	6.36	6.177	66.44	18,645

From Table 1, it's evident that, on average, the age of the participants was 49.90 years (SD = 17.81), with ages ranging from 19 to 85 years. Gender distribution indicated that 58% of the sample were female. Regarding racial and ethnic composition, participants represented a diverse background, with the race variable coded from 1 to 5 (mean = 2.75, SD = 1.15), encompassing Mexican American, other Hispanic, non-Hispanic White, non-Hispanic Black, and non-Hispanic Asian categories.

The overall prevalence of self-reported hypertension was 27%, as indicated by a binary variable with a mean value of 0.27. Psychological factors showed moderate levels across the sample. The mean generalized anxiety disorder (GAD) score was 4.76 (SD = 0.61), while the mean depression score was 4.12 (SD = 1.09). Stress, measured by a proxy variable reflecting irritability lasting two weeks or more, was reported by 57% of participants (mean = 0.57).

Behavioral and anthropometric covariates were also summarized. The mean physical activity score was 2.04 (SD = 0.86) on a scale ranging from 1 to 9, suggesting generally low-to-moderate levels of average daily activity. The average body mass index (BMI) was 27.72 kg/m² (SD = 6.36), indicating that the average respondent was in the overweight range. The number of respondents included in the analysis varied slightly by variable due to missing data, with sample sizes ranging from 18,395 to 18,698 for key variables.

Multivariable logistic regression of hypertension (HTN) on mental health and demographic factors

To examine the independent associations between psychological factors and hypertension, a multivariable logistic regression model was conducted, as shown in Table 2. The model included anxiety, depression, and stress as primary predictors while adjusting for potential confounders such as age, gender, race/ethnicity, physical activity, and BMI.

**Table 2:** Multivariable logistic regression of hypertension (HTN) on mental health and demographic factors. Note: Estimates are based on multiple imputed data with 20 imputations. *p < 0.05, **p < 0.01, ***p < 0.001. Coef: coefficient; SE: standard error; CI: confidence interval; GAD: generalized anxiety disorder score; DEP: depression screening scale; PAQ: physical activity; BMI: body mass index.

	Coef.	SE	p-value	95% CI
GAD	-0.02	0.14	0.88	-0.300, 0.258
DEP	-0.02	0.08	0.83	-0.186, 0.151
Stress: yes	0.30*	0.13	0.03	0.037, 0.567
Age	0.05***	0.00	0.00	0.045, 0.050
Gender				
Female	-0.01	0.04	0.78	-0.090, 0.067
Race				
Other Hispanic	0.18	0.10	0.07	-0.014, 0.376
Non-Hispanic White	0.21***	0.05	0.00	0.116, 0.312
Non-Hispanic Black	0.45***	0.06	0.00	0.332, 0.560
Non-Hispanic Asian	0.14	0.11	0.21	-0.079, 0.355
PAQ	-0.16***	0.02	0.00	-0.207, -0.114
BMI	0.09***	0.00	0.00	0.081, 0.093

The findings from the multivariable logistic regression model in Table 2 assessed the association between mental health indicators, generalized anxiety, depression, and stress and the likelihood of self-reported hypertension, adjusting for demographic and health-related covariates.

The findings reveal that among the psychological variables, only stress, measured via self-reported irritability, was significantly associated with hypertension. Individuals who reported feeling irritable most of the time for two weeks had significantly higher odds of hypertension (β = 0.30, p < 0.03). The 95% confidence interval (0.037, 0.567) indicates a modest but meaningful positive association. In contrast, both anxiety and depression scores were not significantly associated with hypertension. The GAD score showed a coefficient of -0.02 (p = 0.88), and the depression score had a coefficient of β = -0.02, p < 0.83, indicating no evidence of an independent relationship after controlling for other factors.

Several covariates used as control variables were significantly associated with hypertension. Age was strongly and positively associated with hypertension (β = 0.05, p < 0.001), as was body mass index (BMI) (β = 0.09, p < 0.001). Physical activity was inversely related to hypertension (β = -0.16, p < 0.001), suggesting that higher activity levels may offer a protective effect. In terms of race/ethnicity, non-Hispanic White (β = 0.21, p < 0.001) and non-Hispanic Black (β = 0.45, p < 0.001) participants had significantly higher odds of hypertension compared to the reference group (Mexican American). The association for other Hispanic participants approached significance (β = 0.18, p = 0.07), while the coefficient for non-Hispanic Asian participants was not statistically significant (β = 0.14, p = 0.21). Gender was not a significant predictor (β = -0.01, p = 0.78).

## Discussion

This study evaluated the relationship between psychological factors, such as stress, anxiety, and depression, and the prevalence of hypertension in a large, diverse adult population. The findings contribute to the growing body of evidence on the impact of mental health on cardiovascular outcomes. Among the psychological variables assessed, stress showed a statistically significant positive association with hypertension. The findings of our study align with national trends observed in U.S.-based research on anxiety and depression. Our study found a higher prevalence of psychological distress in younger adults aged 18-39, consistent with data showing elevated rates of anxiety and depression in this demographic [13,14]. Furthermore, studies among college students reported anxiety and depression rates exceeding 30%, paralleling the trends seen in our dataset [15,16]. These similarities indicate that changes in life and school stress might lead to emotional challenges, highlighting the importance of specific mental health support to reduce related heart risks like high blood pressure. Individuals who reported feeling irritable most of the time over two weeks had higher odds of having hypertension, even after adjusting for demographic and lifestyle covariates. This finding supports prior research suggesting that emotional strain and stress-related responses can influence blood pressure regulation through both behavioral and physiological pathways. Mindfulness-based therapy provides hypertensive patients with depression and/or anxiety a tool to deal with their negative feelings and physical symptoms through a relaxing approach, perhaps by increasing their threshold or reducing their sensitivity to problems [17].

In our study, however, anxiety and depression were not significantly associated with hypertension when analyzed using the multivariable model. While earlier studies found different links between these conditions and blood pressure, the lack of significant results in our study might be because of complicated relationships with other factors like body weight, physical activity, and age. It’s also possible that the overlap between mental health issues weakened the separate impacts of anxiety and depression when looked at alongside stress and other influences. Furthermore, the shared variance between mental health conditions may have diluted the independent effects of anxiety and depression when modeled together with stress and other predictors. This analysis confirmed established risk factors for hypertension. Older age and higher BMI were both significantly associated with increased odds of hypertension, while higher physical activity levels were linked to lower odds. These results are consistent with current epidemiological knowledge and highlight the continued importance of addressing lifestyle behaviors in hypertension prevention. Similarly to our work, disparities in differences between different races and how they react to stress and its attendant hypertension are well documented and likely reflect a combination of genetic, environmental, and structural determinants of health [5,18].

Strengths and limitations

Our study’s primary strength is the large, diverse, and nationally representative NHANES sample, which enhances external validity and provides substantial statistical power. We also employed multiple imputation by chained equations (MICE) with 20 imputations and diagnostic checks to address substantial missingness, preserving our sample size and reducing potential bias. However, several limitations warrant consideration. The cross-sectional descriptive design prevents causal inference and raises the possibility of bidirectional or reverse causality (e.g., hypertension contributing to stress). Approximately 80% of data for mental health variables were missing, and although the MAR assumption under MICE was supported by diagnostics, it may not fully hold. In the absence of formal stress scales, such as the Perceived Stress Scale (PSS), we used self-reported irritability as a proxy, which may overlap with anxiety and depression constructs and fail to capture the full complexity of stress. Self-reported hypertension may introduce misclassification based on recall or prior medical diagnosis. Finally, other psychosocial distress indicators, insomnia, social isolation, and perceived stress, were not available in these NHANES cycles, limiting our ability to assess their potential influence on hypertension.

Future research should employ longitudinal designs with comprehensive, validated psychosocial instruments to elucidate temporal and causal pathways between psychological distress and hypertension.

## Conclusions

This study examined the association between psychological factors, stress, anxiety, depression, and hypertension in a large adult population. Among these factors, stress was found to be significantly associated with increased odds of hypertension, even after adjusting for demographic and lifestyle variables. In contrast, anxiety and depression did not show significant independent effects. These findings emphasize the potential role of emotional strain in the development or persistence of hypertension and highlight the importance of incorporating psychological well-being into hypertension risk assessments. This differential finding may be due to stress-related irritability capturing acute physiological arousal (e.g., HPA-axis activation and sympathetic response) more directly than symptom-based scales for anxiety or depression. Additionally, overlapping variance among mental health constructs in multivariable models could attenuate the distinct effects of anxiety and depression. Traditional risk factors such as age, BMI, and physical inactivity were also significantly associated with hypertension, reaffirming their critical role in cardiovascular health. In addition, racial disparities in hypertension prevalence were observed, consistent with long-standing evidence on health inequities across ethnic groups.
